# Managers’ experience of success criteria and barriers to implementing mobile radiography services in nursing homes in Norway: a qualitative study

**DOI:** 10.1186/s12913-018-3115-9

**Published:** 2018-04-25

**Authors:** Elin Kjelle, Kristin Bakke Lysdahl, Hilde Merete Olerud, Aud Mette Myklebust

**Affiliations:** 1 Department of Optometry, Radiography and Lighting Design, Faculty of Health and Social Sciences, University College of Southeast Norway, Postboks 235, 3603 Kongsberg, Norway; 2 Institute of Radiography and Dental technology, Department of Life Sciences and Health, Faculty of Health Sciences, Oslo and Akershus University College of Applied Sciences, Postboks 4, St. Olavs plass, 0130 Oslo, Norway

**Keywords:** Mobile radiography service, Barriers, Facilitators, Implementation, Coordination, Nursing homes, Mobile health units, Radiography, Telemedicine

## Abstract

**Background:**

In order to meet the future challenges posed by ageing populations, new technology, telemedicine and a more personalized healthcare system are needed. Earlier research has shown mobile radiography services to be highly beneficial for nursing home residents in addition to being cost-effective. Despite the benefits, mobile radiography services are uncommon in Europe and Norway. The purpose of this study was to explore success criteria and barriers in the process of implementing mobile radiography services, from the point of view of the hospital and municipal managers.

**Methods:**

Eleven semi-structured interviews were conducted with managers from five hospitals and six municipalities in Norway where mobile radiography services had been implemented. Core issues in the interview guide were barriers and facilitators in the different phases of implementation. The framework method for thematic analysis was used for analysing the data inductively in a research team.

**Results:**

Five main categories were developed through the success criteria and barriers experienced by the participants: national health policy, regional and municipal policy and conditions, inter-organizational implementation projects, experienced outcome, and professional skills and personal characteristics. The categories were allocated into three higher-order classifications: macro, meso and micro levels. The main barriers experienced by the managers were financial, procedural and structural. In particular, the reimbursement system, lack of management across healthcare levels and the lack of compatible information systems acted as barriers. The main facilitators were external funding, enthusiastic individuals in the organizations and good collaboration between hospitals and municipalities.

**Conclusions:**

The managers experienced financial, structural and procedural barriers. The main success criteria in the process were external funding, and the support and engagement from the individuals in the organizations. This commitment was mainly facilitated by the intuitive appeal of mobile radiography. Changes in healthcare management and in the financial system might facilitate services across healthcare levels. In addition, compatible information systems across healthcare levels are needed in order to facilitate the use of new technology and mobile services.

**Electronic supplementary material:**

The online version of this article (10.1186/s12913-018-3115-9) contains supplementary material, which is available to authorized users.

## Background

The use of telemedicine, new technology and a personalized healthcare system are some of the measures used to meet the challenges of ageing populations in Europe [1]. One of the concerns is an increase in the number of persons living in nursing homes [2]. More than 80 % of nursing home residents have dementia and many are living with several comorbidities [3]. Thus, nursing home residents need highly coordinated and integrated services [4]. In addition, there is a high incidence of infections and injury due to falls [5–7]. All these elements increase the need for specialized healthcare services, including imaging service, among nursing home residents compared to the rest of the population [3, 7]. According to Wang et al. [7] about 72 % of nursing home residents visiting an emergency department need an imaging test and 85 % of these tests are conventional x-ray examinations. Today, radiological services for nursing home residents are usually provided at a hospital or in an emergency room. New surroundings such as an x-ray department may increase the risk of falling and/or developing anxiety or delirium [7–10]. Mobile radiography as a telemedicine service allows residents to stay in the nursing home, which may reduce these kinds of complication and avoiding admission to hospital [11, 12]. Recent research suggests that mobile radiography services in nursing homes are psychologically preferable for the residents and that these services are also economically preferable [12, 13]. Despite these advantages, mobile radiography services are not common in Europe. In Norway, only five out of twenty public hospital trusts and one private hospital are in the process of implementing or have implemented mobile radiography services through implementation projects [12]. Oxman et al. [14] list a number of barriers to coordination or integration in healthcare systems, based on Nolte and McKee [15]. These barriers are structural, financial, legal, procedural, professional, conflicting views, organizational self-interest, organizational turbulence and introduction of a competitive environment [16]. To overcome these barriers and succeed in implementing an innovation or change, both organizations and individual professionals need to have a common understanding of the problem, and agree that change is needed [14, 16]. For an easier implementation process the innovation needs to be considered to have a low financial risk. In addition, the innovation or change needs to be considered useful, compatible with existing values, easy to use and to have a large number of supporters in the organizations [14, 16]. During the process of implementation, evaluation of the impact of the innovation on the organization is important. In addition, this impact must be communicated to the individuals in the organization [16]. Further, adequate finances for training and for building the structure are important during implementation in order to succeed.

This study forms part of a larger research project on mobile radiography services for nursing home residents in Norway. The objective of this study was to identify success criteria and barriers in the process of implementing mobile radiography services, from the hospital and municipal manager’s point of view. This knowledge could make it easier for other hospitals and municipalities to implement mobile radiography services and similar services.

Accordingly, this study addressed the following research questions:

- What do managers in municipalities and hospitals experience as success criteria in the implementation of mobile radiography services?
- What do managers in municipalities and hospitals experience as barriers to implementing mobile radiography services?

## Method

This is a qualitative study based on semi-structured interviews. Before describing the methods in detail, the study context is presented.

### Context: The Norwegian healthcare system

The Norwegian healthcare system is mainly a public system based on general taxation. The system is managed politically at the ministry and municipality levels [17]. The municipalities mainly provide primary healthcare services including general practitioner services (GPs), preventive care, nursing homes and rehabilitation [17]. Specialized healthcare, including imaging services is mostly provided by hospital trusts, led by the Ministry of Health and Care Services through regional health authorities [17]. The Norwegian healthcare system struggles with fragmentation challenges due to the lack of central responsibility for coordination across services [14]. To meet these challenges, the Coordination Reform was implemented in Norway in 2012. The aims of the Coordination Reform were to improve public health and the quality of health services in a sustainable manner [18]. In the process of implementing the Coordination Reform, municipalities could apply for funding for collaborative projects that aimed to transfer tasks from the hospitals to the municipalities [19]. In general, health and care services are funded by a combination of block grants, activity-based financing and patient fees [18].

### Participants and local context

Semi-structured interviews were conducted with eleven mangers from five hospitals and six municipalities, which is an acceptable sample size for studies that utilize a qualitative approach [20].

The participants in this study were recruited from municipalities and hospitals where mobile radiography services had been implemented during the last decade. Using volunteer sampling [21], participants from different healthcare levels and management levels were included. All participants volunteered by contacting researcher EK after an invitation was sent to the municipalities and hospitals defining the inclusion criterion. The inclusion criterion was: managers who had been involved in the implementation of mobile radiography services. In municipalities this included either administrators of health services, or managers of nursing homes. In hospitals this included either departmental (x-ray department) or directorial (higher order) managers. Further information about the participants and their context are presented in Table 1. Participants were provided with an information letter and consent form via e-mail when volunteering. The signed consent form was collected by EK before starting the interview.

**Table 1:** Information about the participants and the local context

Area	Number of municipalities included in the service	Population in the area [31]	Size of the area (km^2^) [32]	Participant	Management position	Experience as a manager^a^
A	10	369,714	1823	1	Hospital administrator^b^	Long
				2	Municipal administrator^c^	Long
B	2	135,248	694	3	Municipal administrator^c^	Long
				10	Manager of x-ray department^e^	Long
C	10	230,899	2174	4	Manager of nursing home^d^	Long
				7	Manager of x-ray department^e^	Long
D	8	212,109	2004	5	Manager of nursing home^d^	Long
				6	Manager of nursing home^d^	Short
				11	Manager of x-ray department^d^	Long
E	5	315,462	1402	8	Municipal administrator^c^	Long
				9	Project manager at the hospital^f^	Just for the project

^a^Short experience is defined as less the two years, long experience is defined as more than two years

Exact time in management position was not part of the data collection

^b^Managers working in administrative positions outside the x-ray department

^c^Managers working in administrative positions in municipal administration

^d^Managers working in a nursing home department with personnel management

^e^Managers working in an x-ray department with personnel management

^f^Project manager for the local mobile x-ray service project

All mobile radiography services that were included were implemented through collaborative implementation projects with one hospital and several municipalities. The number of municipalities covered by the services varied from 2 to 10. In all areas, health cooperation committees between municipalities were in place prior to the implementation of mobile radiography services. Three areas (B, D and E) had completed the mobile radiography service project, and mobile radiography had become part of the regular imaging service. The rest of the projects were in their last year and were in the process of including mobile radiography as part of the regular service.

### Data collection

The semi-structured approach was chosen to ensure that the same topics were discussed with all the participants whilst allowing relevant topics to be explored when they arose [20]. An interview guide with open-ended questions was developed for the core issues to be discussed openly. Core issues included barriers and facilitators for the decision to implement mobile radiography services in the implementation process and in daily practice. The interview guide is available in Additional file 1: Table S1. The interviews were conducted from February to May 2016, and were carried out at a place of the participants’ choice, all at the participant’s work place. A Zoom H1 Handy Recorder was used to record the interviews. EK interviewed all the participants and researcher AMM was present at three of the interviews (interviews one, four and seven). The interviews lasted on average 40.9 min (17.3–53.2 min). In order to reach consensus [20], at the end of each interview, EK summarized the participant’s statements of the main subjects in the interview.

The Norwegian Centre for Research Data approved the handling and storage of personal information in this study. The Norwegian Centre for Research Data considered approval of this study by the Ethical Committee to be unnecessary.

### Analysis

The analysis took place in a team of four members (the authors) and used the framework method for thematic analysis, as described by Ritchie [22]. The use of the framework method is a well-established approach in thematic analysis of semi-structured interviews [22]. The analysis process of the framework method in a team has seven stages, as described by Gale et al. [23]. The way in which these stages were applied in the study is presented in Table 2.

**Table 2:** The seven stages of the analysis process used in this study based on Gale et al. [23]

Stage	Who and how
Transcription	EK transcribed the recordings verbatim
Familiarization	All researchers familiarized themselves with the data by reading through the transcripts
Coding	Two researchers coded openly three transcripts
Developing the working analytical framework	All 4 researchers met to discuss and agree on a set of codes grouped into categories in a working analytical framework. In addition, the researchers met to engage in reflexivity discussions. Using this analytical framework and further open coding, two researchers coded the next four transcripts. Then, after a new discussion, the team revised and refined the framework. This process was repeated until all transcripts were coded. Finally a final thematic framework was agreed upon. The framework consisted of six categories with 4–10 codes each. These are available in Additional file 1 Table A2.
Applying the analytical framework	EK indexed all transcripts using QSR International’s NVivo Pro version 11 software (NVivo).
Charting the data into the framework matrix	EK charted the data into 33 matrices consisting of cases in the rows and codes in the columns using NVivo.
Interpreting the data	Thematic analysis for descriptive purposes was performed by EK supported by AMM, as described by Ritchie [24]. Elements were detected in the data summaries in the matrices. These elements were sorted according to underlying dimensions and key dimensions were identified. Then categories and higher order classifications were identified. All the researchers met to discuss findings and to reach a consensus.

### Trustworthiness

As recommended by Yardley [24] the initial analysis was determined through discussions and consensus in the team performing the analysis. Transparency and coherence are supported by the audit trail in the framework method [23]. This is ideally evident in the close correspondence between the presented data and the claims made. Impact and importance were tested by presenting this study at two open research seminars. Feedback from these seminars was used to refine the analysis.

## Results

**Fig. 1:**
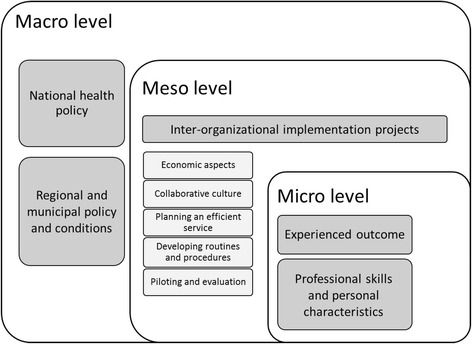
Classifications, categories and sub-categories from inductive data analysis. The 3 higher-order classifications are represented by the white boxes. Categories are represented by dark grey boxes within each level. In the meso level there are sub-categories represented by light grey boxes

Eighty key dimensions were identified in the matrices developed in QSR International’s NVivo Pro version 11 software (NVivo) (see Additional file 1: Table S3), these were allocated into five categories: national health policy, regional and municipal policy and conditions, inter-organizational implementation projects (including five sub categories), experienced outcome, and professional skill and personal characteristics. These main categories were allocated into three higher order classifications: macro, meso and micro levels. The classifications, categories and sub-categories are presented in Fig. 1.

The category inter-organizational implementation projects at meso level was considered a core category where the subcategories describe a variety of factors affecting the actual implementation process. As the respondents act on the meso level, the data are more detailed in this level. First the macro and micro levels will be presented with barriers and facilitators affecting the internal processes and measures taken in the meso level.

### Macro level

#### National health policy

The Norwegian healthcare system influenced the implementation of mobile radiography services in different ways, both as a barrier and a facilitator. Financial barriers and facilitators were most prominent. Managers experienced that the reimbursement system for specialized healthcare services was made for services provided in hospitals. The fee-for-service reimbursement for an x-ray examination was identical whether the service was provided in a hospital or in a nursing home. This was considered unfair by both hospital and municipal managers, because the resources used by the x-ray department were significantly higher per examination with the mobile service. Thus, the activity-based part of the funding was a barrier for mobile services. One hospital administrator said:

“*The reimbursement system was not made for mobile services… It was made for an old-fashioned system in which things happen within four walls, such as in hospitals*”.

The participants considered that the Coordination Reform facilitated mobile services by defining the principles for cooperation between hospitals and municipalities through contracts. In addition, the Reform allocated funding for projects. This funding facilitated projects. However, when this funding was no longer available, the participants experienced this as a barrier for new mobile radiography services. One municipal administrator said:

“*The funding facilitated it [mobile radiography services]*”.

### Regional and municipal policy and conditions

Several barriers were identified at the regional and municipal levels. One barrier identified was organizational changes. In large reorganizations there was no time or money for innovation. In addition, both financial and structural barriers were identified. Mobile radiography represented a new way of cooperation and coordination across healthcare levels. Mobile radiography services are not part of ordinary primary care, and general radiography services are not usually mobile. Thus, municipalities or hospitals are not legally obligated to provide these services. This makes a mobile radiography service a different kind of healthcare service, and a new way of organizing and financing services. Thus, in municipalities, support from local politicians was important for money to be allocated in the budget. The process of gaining political support was time consuming and acted as a barrier. One nursing home manager said:

*“This must also be supported at the chief municipal executive level. And when councilors change jobs or when new municipal councils come into power and so on, there is a risk that these kind of projects disappear, because they have such weird financing”*.

### Micro level

#### Experienced outcome

Most staff and managers had a positive attitude towards mobile radiography services. Participants said that a few physicians in nursing homes were sceptical to mobile radiography services because they considered that a clinical diagnosis was sufficient for these residents. So they regarded imaging tests as unnecessary. Most participants experienced that staff and managers considered imaging tests to be an important diagnostic tool for nursing home physicians, and that avoiding transfer to hospital was important for the residents. Mobile radiography was considered to increase the quality of the service for residents and family members. Also, the general quality of care in the nursing home was considered to be improved, because personnel did not need to spend time arranging for volunteers or family members to accompany residents to hospital for an x-ray, or to accompany residents themselves. One nursing home manager said:

*“So here we are working with placing the patient in the centre, and thinking holistically about the patient. And this [mobile radiography] is very patient friendly. And I think that’s an important argument … It [mobile radiography] saves us a lot of frustration in relation to transportation, waiting for an ambulance, and finding someone to accompany the patient. It is much easier when we can just call and they say we’re coming rather than using family members or the volunteer centre”.*

#### Professional skills and personal characteristics

The participants experienced that key personnel initiated, motivated or facilitated the service. These projects were mostly initiated by one person within the hospital or municipality. Without these enthusiasts the service may not have been implemented. One municipal manager said:

*“We had a doctor who was enthusiastic, an elderly physician at the time, who was really into this [mobile radiography] …, she was very motivated and tried to persuade me to say that we had to have this”.*

In the implementation process, the radiographers running the services had an important role in marketing and establishing good relations with the nursing home staff. One nursing home manager said:

*“Those who come here are very nice, very helpful and very welcoming. It mean a lot that those who provide the treatment [mobile radiography] also think that this is a great service”.*

### Meso level

#### Collaborative implementation projects

The mobile radiography service needed to be defined and set up in cooperation between the hospital and the surrounding municipalities. This presented both administrative and practical challenges such as referral routines, communication, parking and adapting the vehicle. All areas established implementation projects led by the hospital, with managers from both the hospital and the collaborating municipalities. These projects involved deciding about purchases, organizing the service, marketing, and evaluating the service. One municipal administrator said:

*“When the decision was made, when we decided, yes we will have a project, and we have the money, then we had to ensure that all the structures were in place first. So we established a project with a steering committee and project group”.*

#### Economic aspects

Because the idea to implement mobile radiography services mostly came from one enthusiast, getting support from the top management in all the organizations was the greatest challenge. This support was necessary in order for money to be allocated in the budget in the organizations. One municipal administrator said:

*“Perhaps the challenge with this kind of project was that it came from an enthusiast. It lacked anchorage in top management. It was a good project and it was nice to talk about it. But the lack of anchorage in top management made the funding a challenge”.*

Because of the financial barriers at the macro level, most managers in hospitals and x-ray departments were not willing to invest in equipment, a vehicle and staff for the mobile service. The risk was considered high, because this was a new type of service that there was limited knowledge about and little experience of its use and efficiency. To overcome the financial barriers most of the projects applied for external funding and used contracts between the hospital and municipalities, as recommended by the Coordination Reform. This provided financial security and divided the costs between all the parties. The most common financial model used was one where the hospital covered 50% of the costs and the participating municipalities covered the other half of the costs. The division among the municipalities was usually calculated based on the number of inhabitants in the municipality. One hospital administrator said:

*“For the part that was not externally funded, we agreed on a 50-50 economic model. The municipality covered half of the costs and the hospital trust covered the other half… This was really important for the hospital trust”.*

However, developing and agreeing on contracts took a lot of resources and slowed down the implementation. In one area, the bureaucratic process of contracts was the main reason for not involving the municipalities financially. The manager from the x-ray department in this area said:

“*If we had contracts with several municipalities, the contracts would need to be revised and kept up to date. We were not actually talking about much money, so the disadvantages of the bureaucracy outweighed the benefits”.*

#### Collaborative culture

The participants from the municipalities experienced the x-ray department managers as respectful and grateful. All project members were highly committed and engaged in the project. Participants described a good collaborative culture within the project groups. They all wanted to increase the quality of the services for residents. Thus, they kept working despite the barriers. One nursing home manager said:

*“You must have enthusiasm all the way, if not you will fail”.*

In the area where the hospital covered all costs, cooperation was also important, not in order to gain support from the management in the municipalities, but to understand the needs of the nursing homes. The x-ray department manager in this area said:

*“It was very important to involve them [the nursing home staff]. They could point out their needs and the importance of having x-ray as a diagnostic tool in the nursing home. I think that was very important”.*

#### Planning an efficient service

Another important aspect in the implementation project was to tailor the service to the local demographics to ensure efficiency. Travel distances and traffic in the area were considered when planning the services. The population size needed for the service to be cost-effective was perceived differently in the different areas. In one area, two municipalities with just over 130,000 inhabitants was considered sufficient. However in another area, more than 300,000 inhabitants was considered to be necessary for the project to be cost-effective. However, all participants agreed that the service needed to be in an urban area. One hospital administrator said:

*“This is a typical example of mobile services being cost-effective in densely populated areas, quite the opposite of what people think”.*

To keep the service running all year with an appropriate response time, a group of radiographers rotating within the service was needed (2–7 in these projects). Keeping the service up and running was important for the quality and reputation of the service. If the services failed to arrive on time, the nursing homes would send residents to a hospital instead. In all areas the service was available on weekdays, daytime only. It was considered important that the examinations were done within the next day, because most examinations were semi-acute. One x-ray department manager said:

*“We’ve said that these are semi-acute examinations. Our aim is to carry out the examination within the course of the next day, but it is not guaranteed, it’s one of those semi-acute services”.*

To initiate the required treatment, the radiologists were required to report the examination on the same day and call the nursing home if there were any findings that required immediate action. If the nursing home physician was present when the examination was carried out, the images could be viewed on site as well. In addition, the radiographers communicated with the physician directly, or a radiologist by phone, if they suspected critical findings (e.g. fractures) in the images during the examination. One municipal administrator said:

*“Yes, if you are at the bedside you get to see the image and that’s ok… we need the results quickly, we get the results mostly the same day. She [the radiographer] looks at the images there and then as well, and lets us know if there is anything special”.*

It was planned to send referrals and reports electronically between the nursing homes and the hospitals. In addition, wireless transfer of images from the mobile unit to the hospital was planned in order to reduce reporting time. However, none of the projects had come so far. They experienced a combination of legal and procedural barriers for wireless image transfer. To avoid these barrier they used paper-based referrals and reports. In addition, they sat up connections for image transfer at places that were easily accessible outdoors in different hospitals, to transfer images via memory stick or cable. One x-ray department manager said:

*“Transfer of images was also a challenge in other projects. We have not yet come so far either. So we are still working on this, and we have the money, but we haven’t got the solution up and running yet… Now we use a memory stick”.*

#### Developing routines and procedures together 

The participants described collaboration in development of a new service, routines and clinical procedures as important. In one area, routines were not discussed with the nursing home physicians prior to piloting the service. This made the implementation a bit chaotic. There were misunderstandings about referral routines and what types of examination it was possible to do in the nursing home. In the other areas, the target population and what types of x-ray examination to offer were discussed in the implementation projects. In addition, what kind of assistance the radiographers needed at the nursing home, and routines for referral, bookings and communication, were discussed. One x-ray department manager said:

*“They [nursing home physicians and nurses] were involved in defining the service and talked about their needs in relation to the type of examinations they envisioned, how cooperation with the radiographer should be in the nursing home and with our radiologists here in relation to the results when we did not have wireless image transfer”.*

#### Piloting and evaluating the service

After the period of planning and getting the equipment and a vehicle in place, the projects started a pilot where just a few nursing homes or municipalities were included. This made it possible to test equipment, to evaluate the facilities in the nursing homes, and for the radiographers to learn how to plan the day efficiently and gain experience in working alone in a mobile service. The x-ray equipment needed to fit safely in the vehicle, and the vehicle needed to be maintained. Further, the x-ray equipment needed to be designed for transportation into and within the nursing home. In addition, there was a need for sufficient power supply in close proximity to the residents’ rooms. One nursing home manager said:

*“So there was talk of testing to be able to deploy this [mobile radiography services] in a sensible way. We tested it in two municipalities first”.*

The pilot was also used for marketing. It was vital to make the service known to the nurses, physicians and managers, and to build networks. It was important to give information to the physicians who were the referrers and the nurses who were the ones who would contact the physician for a medical examination. Otherwise, no-one would use the service. One radiographer was responsible for visiting all the nursing homes to present the service and the new routines. In addition, the service was publicized in the newspapers. One x-ray department manager said:

*“We thought that it was important to have one designated person in the service who would drive around and establish contact with the nursing homes. It was important to have continuous dialogue, to get them to use us [the mobile radiography service]”.*

The participants experienced evaluation during the pilot as important. The projects used feedback and surveys from physicians, nursing home staff and radiographers in their evaluation. In addition, statistics were discussed in the project meetings as a management tool. This would help to increase the use of the service and improve the quality. One x-ray municipal administrator said:

*“We received regular reports from the project manager, showing how much the service was used. This was a good parameter for asking questions in our own organization: Why are we not using mobile radiography? And we could compare ourselves with others. This has been a good tool”.*

In summary, the barriers at the macro level were identified as the national reimbursement system, and large organizational changes. At the meso level, the main barriers identified were gaining support from the top management to get resources for the project with the process of making contracts, lack of management across healthcare levels, lack of electronic communication and wireless image transfer. In order to overcome these barriers, the implementation projects used different measures such as external funding and contracts, piloting, collaboration and manual communication procedures.

## Discussion

**Fig. 2:**
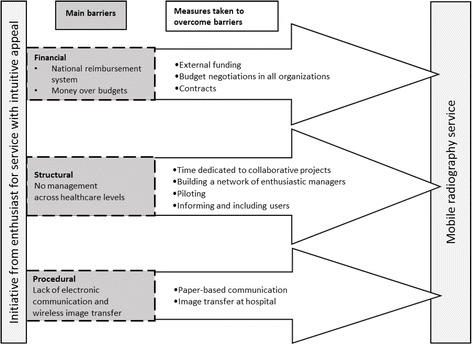
Model of main barriers and measures taken to implement mobile radiography services successfully. The box on the left represents the start of the projects. The dark grey boxes represent the barriers encountered in the implementation. The large arrows represent the measures taken in the projects in order to implement a mobile radiography service

Although there were significant barriers to implementing mobile radiography services, facilitators such as external funding and engaged individuals contributed to overcoming them. The implementation projects adopted different measures to overcome barriers. Although the measures taken were not always ideal, pragmatic solutions were found through piloting and cooperation. The measures taken to overcome barriers in these projects seem to be consistent with earlier research [14, 16] and can thus be transferable to other contexts and technologies. A model of the main barriers and success criteria are presented in Fig. 2.

### Barriers

The barriers encountered were mainly financial, but also structural and procedural. When implementing mobile radiography services, society, municipalities and the hospital may save money because of fewer hospitalizations and less ambulance transport for nursing home residents [12, 13]. However, local managers have a limited overview of the total costs involved in the process. The decision to introduce mobile radiography services is made in the x-ray department where, for example, the reduction in hospitalizations is not visible in the department budget. Thus the economic burden of investment in a vehicle and equipment, as well as engaging new personnel is not offset by the reimbursement system and there is a reluctance to implement mobile radiography services. In order to facilitate telemedicine services, the reimbursement system should have separate telemedicine/mobile codes, with adapted reimbursement as discussed by Kidholm et al. [25]. This could contribute substantially to increasing the number of mobile or telemedicine services. Most of the collaboration projects in this study applied for external funding. External funding was considered to be fundamental to implementation. In the absence of such funding, improvement of the reimbursement system becomes even more important.

Large organizational changes reduce the available time and resources for innovation as well as creating organizational turbulence [14, 16]. In one of the areas included in this study, a large reorganization occurred during the implementation process. The managers used much of their time and resources on reorganization of staff and assignments, leading to the non-prioritizing of innovation and a barrier to innovative processes [14].

Lack of common information systems makes electronic referrals and reporting impossible. Paper-based routines may increase waiting and reporting time. In addition, wireless image transfer was impossible to use efficiently because of the unacceptable transfer time of 45 min per image. Within 45 min the radiographer would mostly be able to return to the hospital for image transfer. Therefore image transfer now takes place in the hospital at the end of the day. The participants were frustrated by the difficulties in setting up electronic communication and wireless image transfer. However, this technical barrier may soon be resolved by infrastructural evolution. Such projects highlight the need for solutions for transfer of referrals and images, and may thereby urge its technological development; progress that could also be useful for other types of mobile services.

After piloting in several areas and countries, other areas should be able to learn from these full scheme pilots, and implement the services more easily. However, experience and knowledge from the pilot schemes is not easily available. Only a few projects have published research from the implementations [9, 11, 26–29], whilst others have written reports, primarily for internal use. In order to facilitate mobile radiography and other mobile services in other areas, knowledge needs to be communicated both nationally and internationally.

### Success criteria

Mobile radiography was considered a financially high-risk innovation by management in hospitals and municipalities, therefore, increasing financial security was an important factor. The review of Greenhalgh et al. [16] indicate that innovations that are considered to be high risk are difficult to implement. All but one area applied for external funding and used contract between the involved parties, facilitated by the Coordination Reform [18, 19]. The use of contract to divide expenses between municipalities and the x-ray department, resulted in long and complicated processes in all included organizations in an effort to increase financial security. This was particularly the case when a large number of parties were involved. However, one hospital found the resources to independently finance mobile radiography services. This avoided the difficult process of achieving agreement with all the participating municipalities. This would appear to be an easier and more efficient way to implement services across healthcare levels.

The establishment of a collaborative implementation project between the hospital and the municipalities was an important facilitator. According to Greenhalgh et al. [16], multidisciplinary project teams working semi-autonomously are associated with the successful implementation of innovations. The fact that the managers in the group met each other with respect and understanding made implementation easier. In all areas the managers were able to visualize the benefits of mobile radiography services for residents, staff and the healthcare service. Mobile radiography services seem to be compatible with the existing values in the organizations involved. This may have helped to create a good collaborative culture within the projects. Greenhalgh et al. [16] stated in their review that agreement about the need for change, and compatibility with existing goals and values in the organization, make the implementation process easier. With such shared understanding and engagement most of the projects developed local procedures and routines in collaboration. Earlier research has shown that adjusting to the local context is an important criteria in success [16].

One important facilitator made by the project groups was networking. Networks can be used for communication and setting up collaboration [16]. The project groups connected managers from municipalities with managers from the x-ray department. However, the use of the group members’ existing networks in their own organizations was just as important. Through these networks, information and feedback between the municipalities and the hospital was conveyed. As shown in earlier research, these networks could help to create a shared understanding of the problem and agreement regarding the best way to organize the service [14, 16].

Piloting made it possible to adapt mobile radiography services to the local context. Piloting and adapting the services are important facilitators to success in an implementation process [14, 16]. During the piloting, the mobile radiography services were monitored and evaluated. In addition, the implications and changes made were communicated to the users in meetings. In the review of Greenhalgh et al. [16] evaluating changes, and keeping individuals in the organizations informed of changes was indicated to facilitate implementation, as involved parties experience an involvement in the process. [16].

Mobile radiography seems to be easily adopted by the users. Generally nursing home and x-ray department personnel thought that transporting residents to hospital for an x-ray was unacceptable/unethical in relation to the negative consequences for the residents. Thus, they shared the impression that mobile radiography services were useful and in the best interests of the residents. This attitude facilitates implementation. Earlier research has shown that a shared understanding of the situation and the need for change makes the individuals adopt changes more easily [14, 16]. To facilitate individual adoption, the projects used meetings, brochures and presentations to educate users about areas of application, benefits for residents, staff and society, and to introduce new routines and procedures.

### Limitations of the study

In this study, recruitment was based on the organizations finding a volunteer to represent them in the interview. To ensure rich data on implementation of mobile radiography services it was important to interview subjects who were included in an implementation project, in accordance with sampling strategies described by Creswell [30]. The participants were all recruited from organizations that were on the way to or had succeeded in implementing mobile radiography services. Thus the sampling may have led to a bias towards participants with a positive experience of mobile radiography projects. However, organizations which have not succeeded in implementing these services in Norway are not known to the authors. In addition, participants were recruited from only a few Norwegian municipalities. Thus, transferability may be difficult in other situations, although, rich, thick descriptions of the setting and participants would enable readers to determine transferability [30]. The results are based on participants’ self-reported data, and no attempts have been made to verify their statements independently.

### Implications for practice

When planning implementation of mobile or telemedicine services, it is important to consider the possibility of the hospitals financing the services. To motivate the hospital management to take responsibility for financing, changes in the macro level seem to be necessary. Developing the reimbursement system or earmarking funds seems appropriate. Further, the healthcare system need managers with responsibility across levels in order to break down boundaries between primary and specialized healthcare. Compatible communication systems are important to increase efficacy when services cross healthcare levels. Thus, regional health authorities should facilitate compatible information systems.

If these changes were made at the macro level, it would be easier for new projects to focus on adaption of the services within the local context, rather than financial contracts. Further, another comprehensive pilot project would be unnecessary at least in the western world. Sufficient evidence on patient safety and utility of mobile radiography services has been established [12, 13]. However, small implementation projects to adapt the services to the local context would help to ensure an appropriate response time, suitable routines and functioning.

### Further research

Further research is needed to explore barriers and success criteria of mobile services in other contexts and for other technologies. In addition, it would be relevant to evaluate the impact of financial barriers separately, as this seems to be the greatest obstruction. Further, evaluating cost-effectiveness of mobile radiography services in more sparsely populated areas of Norway and in other countries would be important in order to be able to recommend a wider implementation of mobile radiography services.

## Conclusion

This study set out to explore managers’ experience of success criteria and barriers to implementing mobile radiography services. The result indicate that financial barriers caused by the financial system were most prominent, along with structural and procedural barriers. In addition to external funding, the main success criteria in the process was the support, engagement and hard work of individuals in the organizations who collaborated in these projects. This commitment was mainly facilitated by the intuitive appeal of mobile radiography services. In order to facilitate more mobile radiography services, or similar services in Norway and other countries, barriers need to be reduced, these include changes in the reimbursement system, or the allocation of earmarked funds. Further, there is a need for compatible information systems and managers with responsibility across healthcare levels.

## Additional file

Additional file 1: Includes the interview guide, analytical framework and identified key dimensions included in categories and higher-order classifications. **Table S1.** Interview guide, **Table S2.** Analytical framework with categories and codes and **Table S3.** Key dimensions, categories and classifications. (DOCX 19 kb)

## Abbreviations

GP: General practitioner
