# Supplementary material for: Managers’ experience of success criteria and barriers to implementing mobile radiography services in nursing homes in Norway: a qualitative study
Source: BMC Health Serv Res. 2018 Apr 25;18:301. doi: 10.1186/s12913-018-3115-9 (PMC5921415; doi:10.1186/s12913-018-3115-9)
Supplement: Supplementary file 1 — Includes the interview guide, analytical framework and identified key dimensions included in categories and higher-order classifications. Table S1. Interview guide, Table S2. Analytical framework with categories and codes and Table S3. Key dimensions, categories and classifications. (DOCX 19 kb) [file 12913_2018_3115_MOESM1_ESM.docx]

**Contents - Additional file**

Table A1: Interview guide

Table A2: Analytical framework with categories and codes

Table A3: Key dimensions, categories and classifications.

Table A1: Interview guide

| **Core questions** | **Major prompts** |
| --- | --- |
| First, could you please to tell me about the process of deciding to introduce mobile x-ray services?  How do you consider the decision process now in hindsight? | - Who took part in the decision-making? - Which role did you take in this process? - Which topics were discuss in this process? - What complicated the decision? - What made the decision process easier? |
| How did the introduction om mobile radiography services proceed? | - What complicated the introduction? - What made the introduction easier? |
| How is the mobile radiography service today? | - What are the challenges? - What facilitates continuation of mobile radiography services? |

Table A2: Analytical framework with categories and codes.

| **1 Background** | |
| --- | --- |
| 1.1 | Details on the participant |
| 1.2 | Work place |
| 1.3 | Existing cooperation groups |
| 1.4 | Status of the project to day |
| 1.5 | Area covered by the service |
| 1.6 | Financial model |
| **2 Frameworks and external factors** | |
| 2.1 | Demographical aspects |
| 2.2 | Financial aspects nationally |
| 2.3 | Law |
| 2.4 | Organisational framework of the health care services |
| 2.5 | Political anchoring |
| 2.6 | Funding |
| **3 Knowledge on mobile radiography, development and innovation** | |
| 3.1 | Experience from mobile radiography and other cooperation projects. |
| 3.2 | Research and documentation |
| 3.3 | Development of technology and the mobile radiography service |
| 3.4 | Attitude towards the service |
| 3.5 | Patient groups and clinical conditions |
| 3.6 | Quality and outcome |
| **4 Collaboration** | |
| 4.1 | User involvement |
| 4.2 | Information and communication |
| 4.3 | Local anchoring |
| 4.4 | Marketing |
| 4.5 | Cooperating and cooperation culture |
| 4.6 | Contracts |
| 4.7 | The understanding of the specialist health care service |
| **5 Organising mobile radiography services** | |
| 5.1 | Responsibility, roles and ownership |
| 5.2 | Routines and procedures |
| 5.3 | Proportioning the service to the demographics |
| 5.4 | Practical and ICT challenges |
| 5.5 | Project and piloting |
| 5.6 | Operational stability |
| 5.7 | Process and bureaucracy |
| 5.8 | Enthusiasts and key persons |
| 5.9 | Interest and motivation |
| 5.10 | Personal skills |
| **6 Economy** | |
| 6.1 | Costs |
| 6.2 | Prioritising and commitment |
| 6.3 | Economic model |
| 6.4 | Economic thinking |

Table A3: Key dimensions, categories and higher order classifications.

| **Categorisation with key dimensions** | **Higher order classifications** |
| --- | --- |
| **National health policy:**   - The funding system - Valuation system and financing - Change the system of funding - Policy central and local - Cooperation reform - Cooperation funding - Cooperation reform (contracts). - Privacy law | **Macro level** |
| **Regional and municipal policy and conditions:**   - Attitude to health service (holistic) - Innovation and organization - Municipal processes - Prioritisation of non-statutory services - Access to/continuity doctors - Reorganisation - Unclear responsibilities - Processes in hospital trusts |  |
| **Inter-organisational implementation projects**  *Developing routines and procedures*   - Professional anchoring - Agree on patient groups - Technical limitations - Missing routines - Good routines   *Piloting and evaluation*   - Development of the service (equipment/referrals) - Car and equipment - Radiographers work environment - Facilitation at the nursing home - Importance of marketing - Downtime - Downtime-car and equipment - Project-> operation - Internal communication - Information for next of kin - Anchoring among employees - Daily communication - Documentation (research/evaluation) - Regular follow-up and evaluation   *Collaborative culture*   - The dialog between the parties/ownership to the project. - Project organisation - Anchoring by means of cooperation - Municipal management helps in the development of the service - Collaborative culture - Joint engagement   *Planning an efficient service*   - Adaption to local conditions - Learn from past projects - Positive experiences - Electronic transmission of images and documents - Profitability - Large enough population basis - Geographical area and efficiency - Relieve the ambulance - Response and report time - Vulnerability and staffing - Use   *Economical aspects*   - Anchorage in management - Bureaucracy - Contracts between the parties - Project funding - Financial security - Economic challenges - Economic thinking hospitals/municipalities/communities. - Silo thinking/holistic economic thinking - Attitude to the funding - Municipal costs - Hospital costs - Financial models - Long-term commitment | **Meso level** |
| **Experienced outcome**   - Increased quality (patient, doctors and in general) - Positive attitudes - Advantage of the service - Utility - Negative experiences with radiology at the hospital. - Human costs | **Micro level** |
| **Professional skills and personal characteristics**   - The initiator - Leadership skills - Promoters - Motivation - Radiographers attributes   •     Patient centeredness   - Physicians role - Radiographers role - Reputation |  |
